# Supplementary material for: Structure of Genes Encoding Oxidosqualene Cyclases—Key Enzymes of Triterpenoid Biosynthesis from Sea Cucumber Eupentacta fraudatrix
Source: Int J Mol Sci. 2024 Nov 29;25(23):12881. doi: 10.3390/ijms252312881 (PMC11641436; doi:10.3390/ijms252312881)
Supplement: Supplementary file 1 [file ijms-25-12881-s001.zip › ijms-3310031-supplementary.pdf]

Structure of Genes Encoding Oxidosqualene Cyclases—Key Enzymes of Triterpenoid Biosynthesis from Sea Cucumber *Eupentacta fraudatrix*

Sergey N. Baldaev [1], Viktoria E. Chausova [1], Ksenia V. Isaeva [1,2], Alexey V. Boyko [3], Valentin A. Stonik [1] and Marina P. Isaeva [1,*]

1 G.B. Elyakov Pacific Institute of Bioorganic Chemistry, Far Eastern Branch, Russian Academy of Sciences, 159, Pr. 100 let Vladivostoku, 690022 Vladivostok, Russia; baldaevsergey@gmail.com (S.N.B.); v.chausova@gmail.com (V.E.C.); issaevakssenia@gmail.com (K.V.I.); stonik@piboc.dvo.ru (V.A.S.)
2 Institute of High Technology and Advanced Materials, Far Eastern Federal University, Ajax Bay 10, Russky Island, 690922 Vladivostok, Russia
3 A.V. Zhirmunsky National Scientific Center of Marine Biology, Far Eastern Branch, Russian Academy of Sciences, Palchevskogo Street 17, 690041 Vladivostok, Russia; alteroldis@gmail.com
* Correspondence: issaeva@piboc.dvo.ru

Supplementary Materials

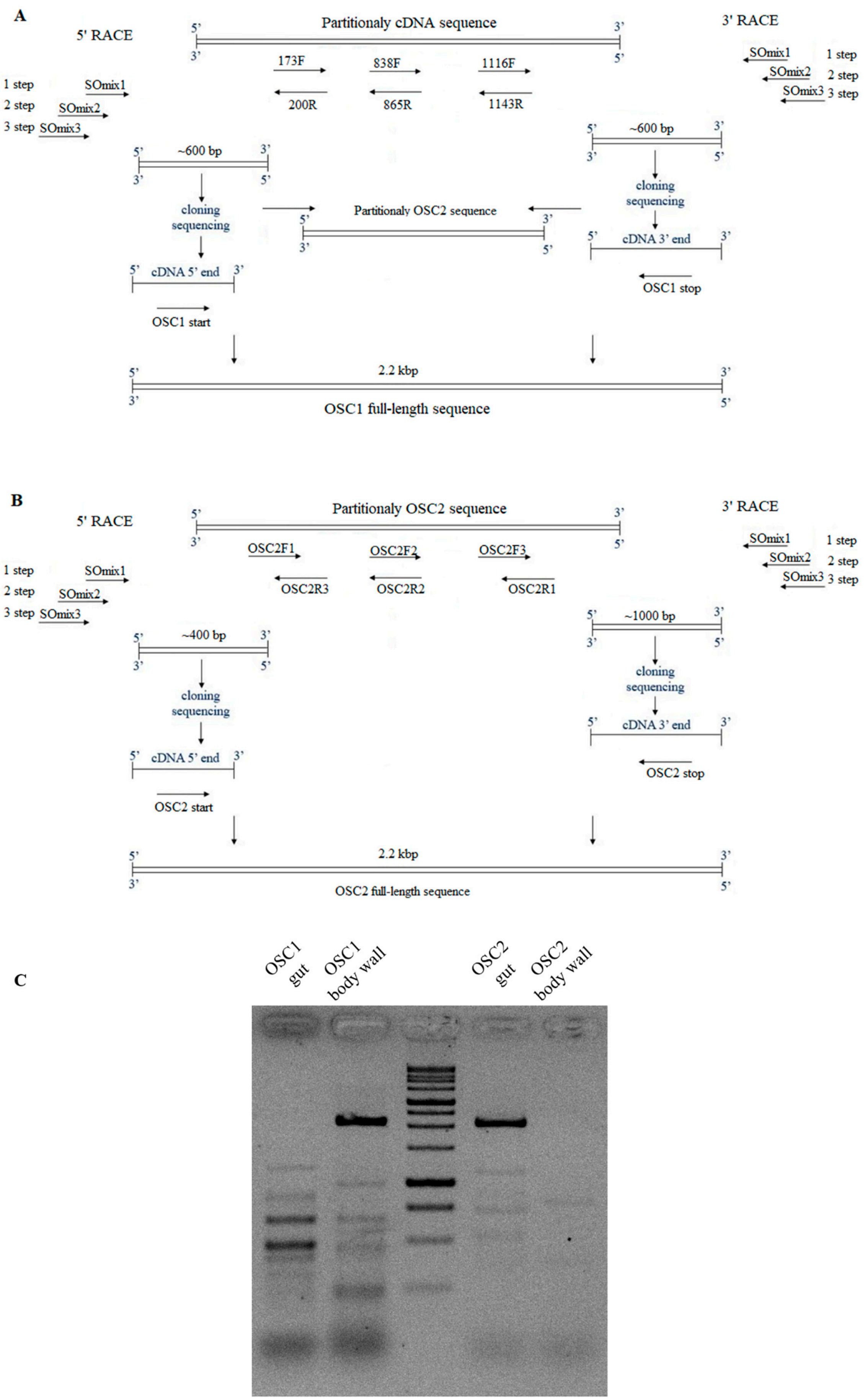

Figure S1. The RACE scheme for cDNA sequencing determination of OSC1 (A) and OSC2 (B) of *E. fraudatrix*; (C) Electrophoresis of OSC1 and OSC2 transcript amplicons.

Table S1. Values of structural alignments of OSCs: 1W6K – *H. sapiens* LSS, LAS1 – *A. japonicus* PS, LAS2 *A. japonicus* LDS, OSC1 – *E. fraudatrix* OSC1, OSC2 – *E. fraudatrix* OSC2.

Pair	RMSD	TM-score	Sequence identity	Matched residues
LAS1-1W6K	1.127	0.977	59.6%	710
LAS2-1W6K	0.928	0.986	57.1%	702
OSC1-LAS1	1.392	0.979	73.1%	706
OSC2-LAS2	1.137	0.980	70.8%	705

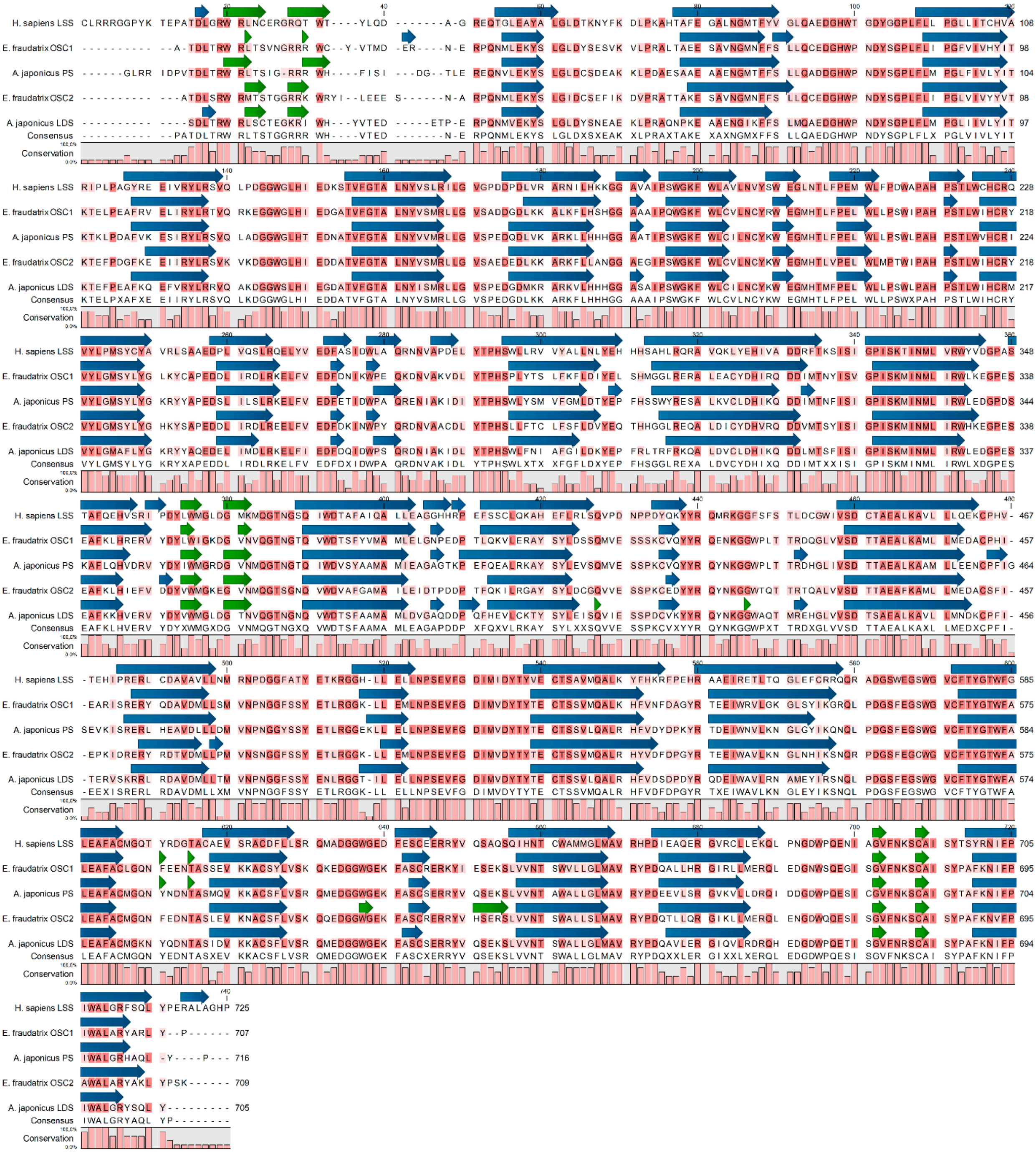

Figure S2. Secondary structure alignment of *H. sapiens* LSS, *A. japonicus* PS, *A. japonicus* LDS, *E. fraudatrix* OSC1, and *E. fraudatrix* OSC2. Secondary structures are shown with blue arrows for alpha helices and green arrows for beta strands.

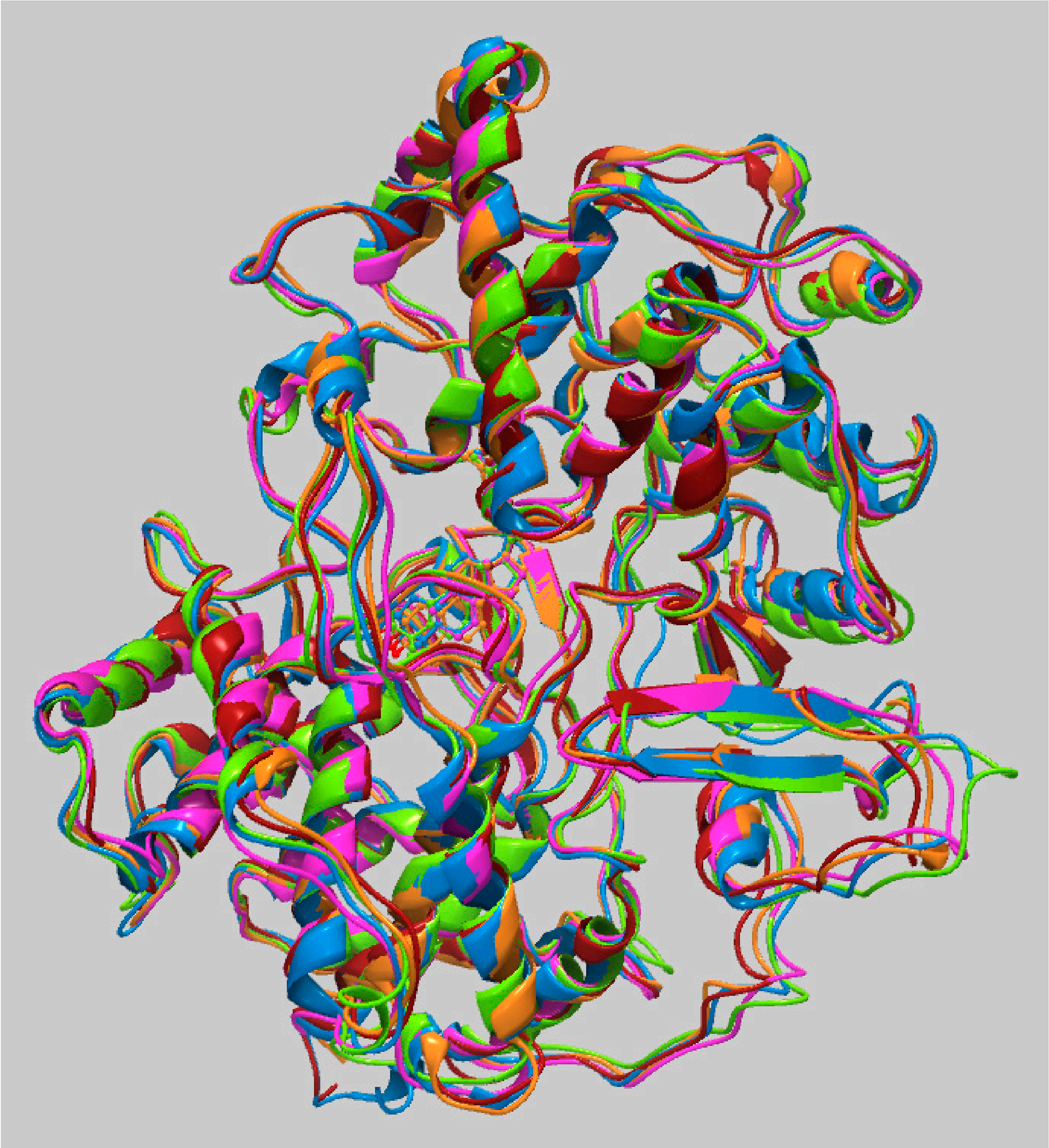

Figure S3. 3D view of OSCs structure superposition: blue – *H. sapiens* LSS; green – *A. japonicus* PS; orange – *E. fraudatrix* OSC1; violet – *A. japonicus* LDS; red – *E. fraudatrix* OSC2.

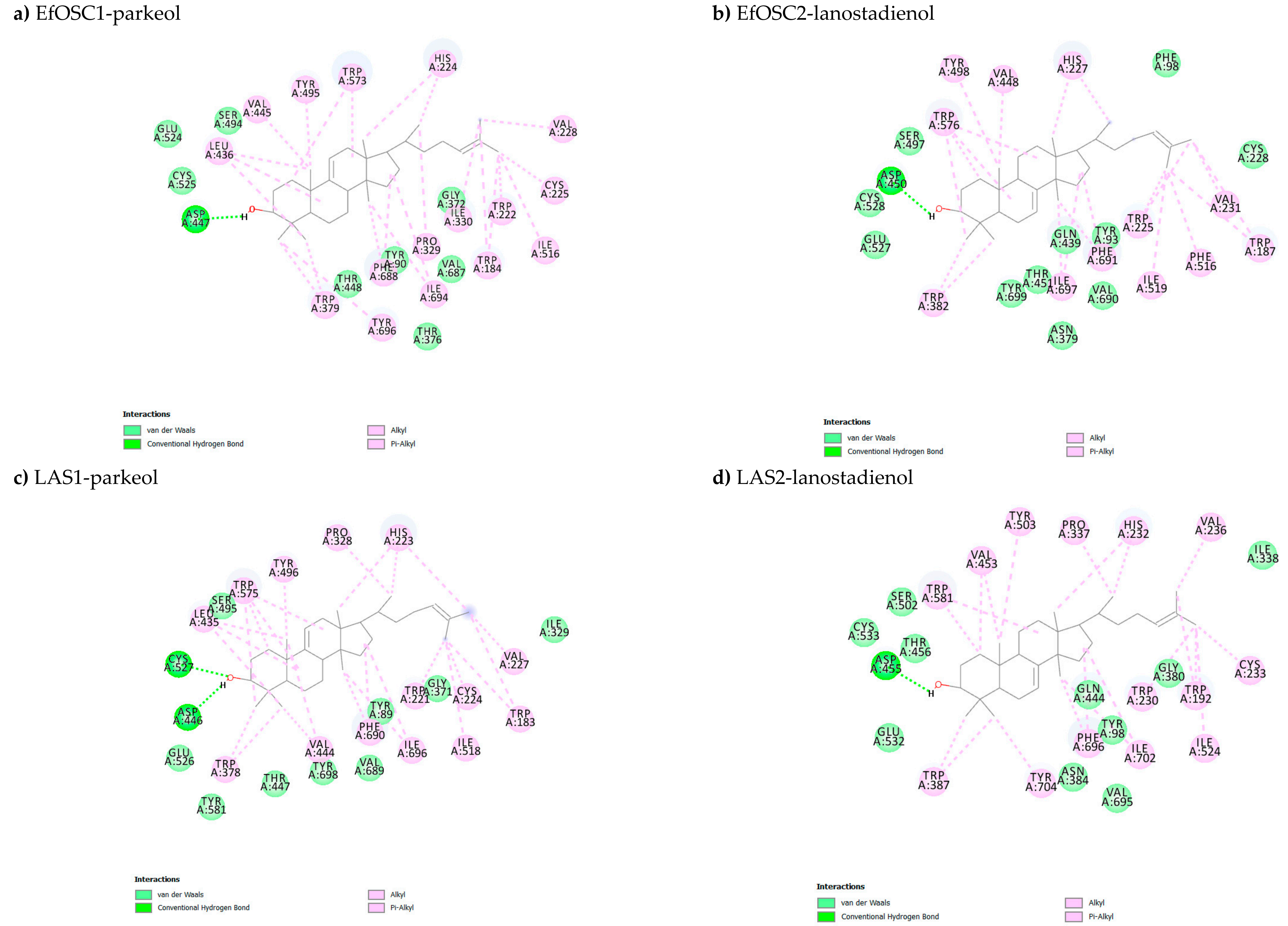

Figure S4. 2D-diagrams of OSCs active center contacts with triterpenoids of parkeol (a,c) and lanostadienol (b,d).

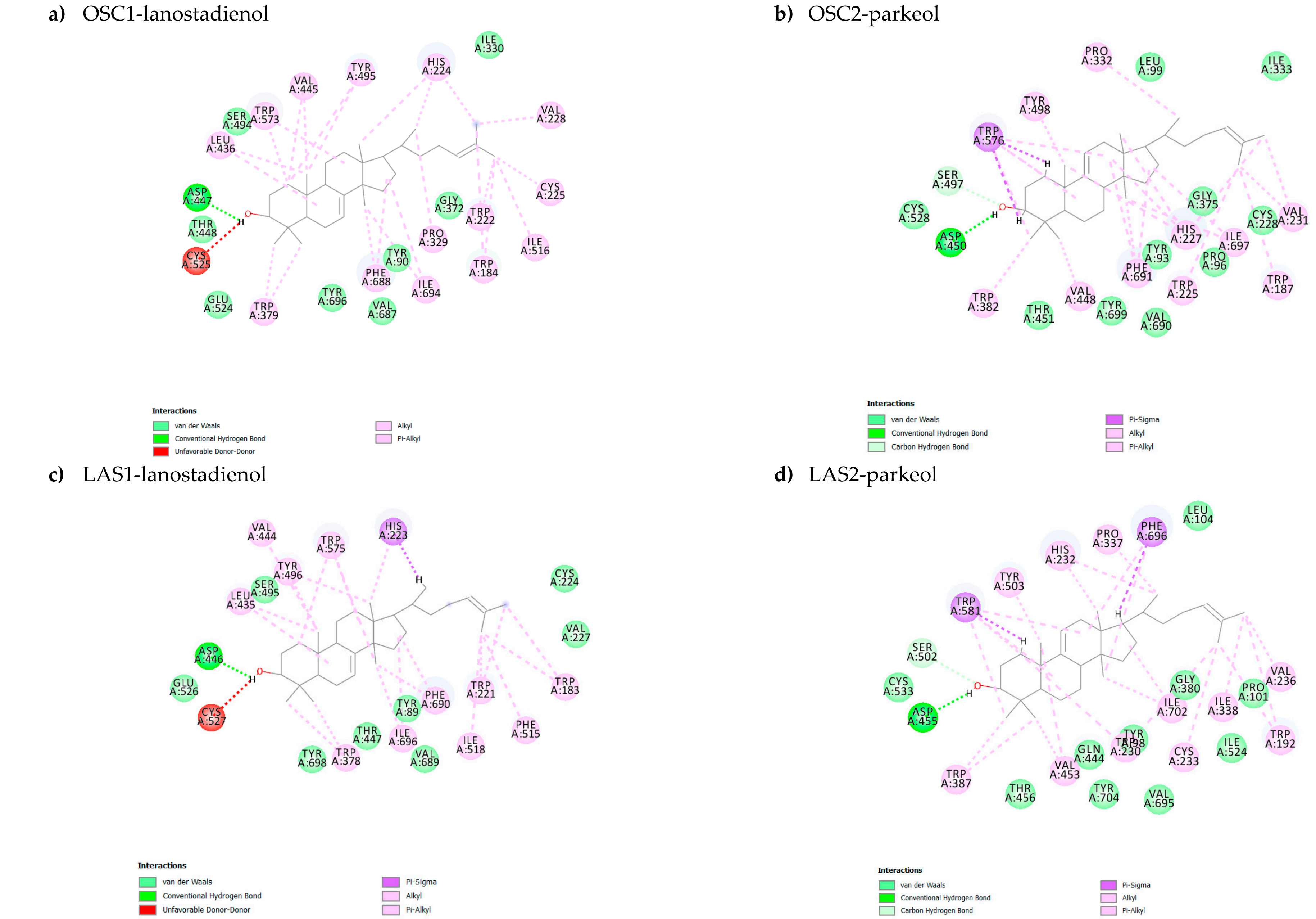

Figure S5. 2D-diagrams of OSCs active center contacts with triterpenoids of lanostadienol (a,c) and parkeol (b,d).

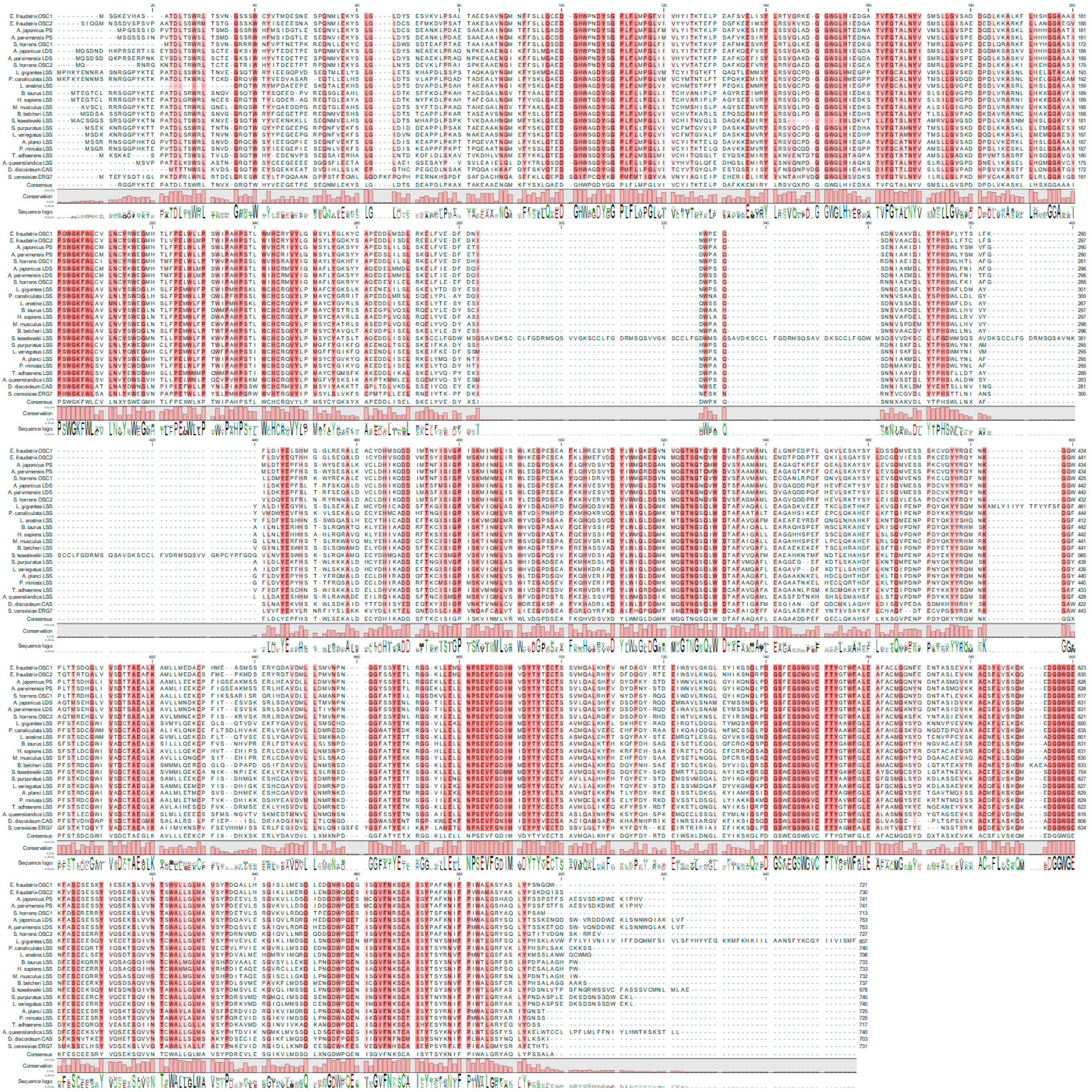

Figure S6. Alignment of OSCs proteins. *H. sapiens* (NM 002340.6), *B. taurus* (NM 001046564.1), *M. musculus* (XM 036155651.1), *B. belcheri* (XM 019790446.1), *S. kowalevskii* (XM 006825036.1), *L. anatina* (XM 013562424.1), *L. gigantea* (XM 009046756.1), *P. canaliculata* (XM 025240299.1), *L. variegatus* (XM 041622296.1), *S. purpuratus* (ON478349.1), *P. miniata* (ON478348.1), *A. planci* (XM 022227483.1), *T. adhaerens* (XM 002110738.1), *A. queenslandica* (XM 003383129.3), *E. fraudatrix* OSC1 (OR725688, this study), *E. fraudatrix OSC2* (OR711403, this study), *A. japonicus* LAS1 (ON478352.1), *A. japonicus* LAS2 (ON478353.1), *A. parvimensis* PS (ON478351.1), *A. parvimensis* LDS (ON478350.1), *S. horrens* OSC1 [H. Liu, 2018], *S. horrens* OSC2 [H. Liu, 2018], *D. discoideum* (XM 641154.1), *S. cerevisiae* (NP 011939.2). Conservative residues are in red colors.
